# Placenta Accreta Spectrum Risk in Endometriosis: A Retrospective Cohort Study with ART Subanalysis

**DOI:** 10.3390/jcm15124684

**Published:** 2026-06-17

**Authors:** Madeline West, Amir Alsaidi, Michael A. Belfort, Hendrik A. Lombaard, Yamely H. Mendez, Christina C. Reed, Amir A. Shamshirsaz, Jessian L. Munoz

**Affiliations:** 1 Paul L. Foster School of Medicine, Texas Tech University Health Sciences Center, El Paso, TX 79905, USA; madeliwe@ttuhsc.edu (M.W.);; 2 Department of Obstetrics and Gynecology, Baylor College of Medicine, Texas Children’s Fetal Center, Houston, TX 77030, USAhennie.lombaard@bcm.edu (H.A.L.); yamely.mendezmartinez@bcm.edu (Y.H.M.); christina.reed@bcm.edu (C.C.R.);

**Keywords:** PAS, endometriosis, ART, outcomes, postoperative

## Abstract

**Background/Objectives**: This study assessed whether endometriosis is associated with an increased risk of placenta accreta spectrum (PAS) disorders and investigated if assisted reproductive technology (ART) further increases the risk in patients with endometriosis. **Methods**: This retrospective study used multi-institutional data from the TriNetX database to identify patients who experienced delivery on or before 31 December 2024, with a prior diagnosis of endometriosis and ART therapy, as coded by CPT and ICD-10 codes. The primary outcomes included up to 7-day perinatal results, such as PAS (accreta, increta, percreta), and maternal complications, including peripartum hysterectomy, transfusion, postpartum hemorrhage (PPH), ICU admission, and sepsis. Risk ratios, 95% confidence intervals, and *p*-values were calculated for endometriosis versus no endometriosis and endometriosis patients with ART versus without ART. **Results**: Out of 3,487,612 patients identified, 24,341 had a prior diagnosis of endometriosis prior to propensity score matching. Propensity score matching was used to control for age, demographic variables, previous procedures, and comorbidities. Compared to controls, endometriosis was linked to a higher risk of PAS disorders (RR 1.74), including accreta (RR 2.22), increta (RR 2.50), and percreta (RR 1.59). Additional complications included peripartum hysterectomy (RR 1.72), transfusion (RR 1.26), PPH (RR 1.35), ICU admission (RR 1.43), and sepsis (RR 1.56). Patients conceived via ART faced greater risks of PAS disorders (RR 2.00), accreta (RR 2.14), hysterectomy (RR 1.63), transfusion (RR 2.10), and PPH (RR 1.66). **Conclusions**: This study shows a positive link between endometriosis and the risk of PAS disorders and maternal complications, and the use of ART in patients with endometriosis further increases this risk, emphasizing the importance of comprehensive counseling and a multidisciplinary approach to delivery planning for this high-risk group.

## 1. Introduction

Placenta accreta spectrum (PAS), which encompasses accreta, increta, and percreta, was previously classified as an invasive placental disorder, with its severity determined by the depth of villous penetration on hysterectomy pathology [1]. This framework was based on hysterectomy specimens and highlighted invasion into the myometrium and beyond on histology [1,2]. However, current models suggest a mechanical failure due to a uterine scar or a myometrial disruption leading to defective decidualization and abnormal placental anchoring at that site [1,2]. These models are shifting the paradigm from a malignancy-like “invasion” model to one driven by tissue injury and repair [1,2,3].

While PAS disorders are considered infrequent, contemporary estimates based on population data suggest an overall prevalence of 0.17% [4]. Still, it may lead to catastrophic maternal morbidity, including hemorrhage, coagulopathy, shock, and death [5]. Furthermore, several well-established risk factors, such as BMI ≥ 30, previous uterine surgery, postpartum hemorrhage, a higher number of prior cesareans, and a placenta previa, further align with the proposed pathophysiologic model of implantation over a myometrial/decidual defect at a scar site [6,7].

Endometriosis is an estrogen-dependent inflammatory disease that affects approximately 10–15% of women of reproductive age and has been linked to abnormal implantation and placentation [8]. Furthermore, existing literature indicates a link between endometriosis and increased rates of placenta previa and postpartum hemorrhage, both well-known risk factors [8,9]. Additionally, emerging evidence suggests a connection between endometriosis and PAS disorders [10]. Moreover, due to the common infertility rates (30–60%) among endometriosis patients, many women conceive with the use of assisted reproductive technology (ART) [11]. However, ART has shown evidence to confer additional, and in some analyses independent, risk of PAS disorders [12,13]. These converging trends raise important clinical questions about whether endometriosis alone increases the risk of PAS disorders and if ART conceptions in patients with endometriosis further heighten that risk. 

Few studies have isolated the link between endometriosis and PAS disorders or maternal complications without considering ART exposure and other confounding factors. Furthermore, many previous studies have focused on the effects of ART on PAS disorders, so the independent contribution of endometriosis to PAS disorder development remains unclear [9,12]. This multi-institutional retrospective cohort study aims to fill this gap by evaluating whether endometriosis independently increases the risk of PAS disorders and peripartum complications, and to determine if conception through ART further raises this risk in women with endometriosis.

## 2. Materials and Methods

### 2.1. Data Source and Study Design

This is a multi-institutional, retrospective cohort study using the TriNetX Research Network, which aggregates real-time, de-identified electronic health record (EHR) data from participating health systems. TriNetX only provides de-identified patient records; therefore, this analysis qualified for exemption from institutional review board oversight under TriNetX publication guidance. The analytic cohort comprised individuals with a documented delivery on or before 31 December 2024. Exposure and outcome ascertainment relied on International Classification of Diseases, 10th Revision (ICD-10) diagnosis codes and Current Procedural Terminology (CPT) procedure codes available within the TriNetX platform. All queries, cohort construction, matching, and analyses were executed within the TriNetX web-based analytics environment. TriNetX normalizes source EHRs across participating organizations; as patient-level identifiers are not available, no external linkage or imputation was performed.

### 2.2. Cohort Assembly and Exposure Definitions

Patients aged 18 years or older with a documented delivery were identified within the TriNetX Research Network. The index pregnancy was defined as the first qualifying delivery event recorded within the database on or before 31 December 2024, using delivery-related diagnosis and procedure codes. Then, patients were stratified based on a recorded diagnosis of endometriosis (ICD-10: N80.x) before pregnancy. Initially, 3,487,612 patients were identified who met the inclusion criteria. Out of them, 24,341 had a prior diagnosis of endometriosis and no previous history of ART before pregnancy.

**Figure 1:**
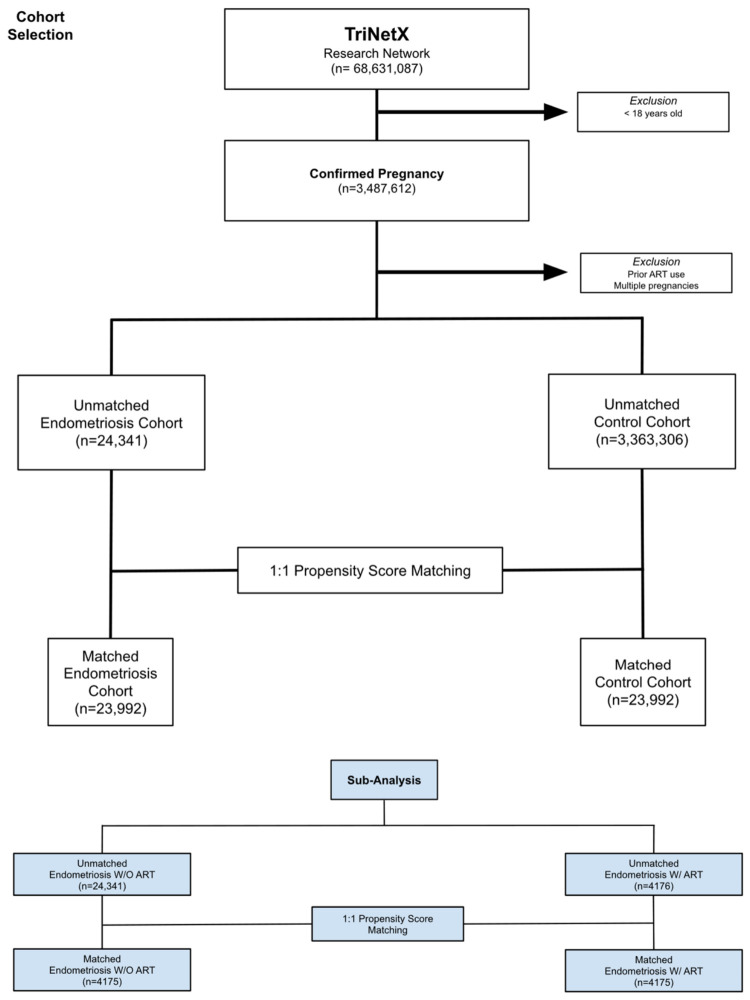
Cohort selection and study design.

Additionally, a sub-analysis was performed comparing patients with a diagnosis of endometriosis. Then, it was stratified into two cohorts based on exposure to assisted reproductive technology (ART). The endometriosis with ART consisted of 4176 patients, while the endometriosis without ART included 24,341 patients. ART utilization was defined by relevant CPT/ICD codes that fall within the umbrella CPT-1012527 code, which included (e.g., oocyte retrieval and embryo transfer procedures: CPT 58970, 58974; laboratory IVF procedures: CPT 89250-89268) and ICD-10 encounter for ART: ICD-10 Z31.83 code associated with the index pregnancy. The study’s design and cohort selection are displayed in Figure 1.

### 2.3. Propensity Score Matching

To reduce the influence of confounding variables, all comparisons underwent propensity score matching (PSM) using the built-in TriNetX analytics platform. In both the primary and sub-analyses, 1:1 propensity score matching was performed using TriNetX’s built-in greedy nearest-neighbor algorithm without replacement to generate cohorts of equal size. Matching parameters, including caliper width, are not user-configurable and are not reported to investigators within the TriNetX platform.

Candidate covariates for the propensity score model included demographic factors such as age at delivery, race, and ethnicity. Additionally, comorbidities and clinical factors including hypertension, diabetes mellitus, leiomyoma, placenta previa, history of uterine procedures, nicotine dependence, and body mass index (BMI) were included in the matching process.

Before matching, significant differences were observed between the cohorts across multiple baseline variables. After matching, both cohorts consisted of 23,992 patients, and standardized mean differences for all matched covariates were <0.01, indicating excellent covariate balance. Results before and after propensity score matching are presented in Table 1 and Table 2.

A similar PSM was applied to the cohort in the sub-analysis. Before matching, both cohorts consisted of 24,341 and 4176 patients, respectively, as shown in Figure 1. The number of patients with Endometriosis and no history of ART, 4176, limited the maximum size of the matched cohorts: after 1:1 propensity score matching, 4175 both cohorts consisted of 4175 patients, and no statistically significant differences were observed between them across the variables.

### 2.4. Outcomes and Statistical Analysis

The primary outcomes included up to 7-day perinatal results, such as PAS disorders overall and individual subtypes (accreta, increta, percreta), and maternal complications, including peripartum hysterectomy, transfusion, postpartum hemorrhage (PPH), ICU admission, and sepsis. For each outcome, estimated risk ratios (RRs) with 95% confidence intervals (CIs) and corresponding two-sided *p*-values using platform-standard methods implemented in TriNetX for matched cohorts. The threshold for statistical significance was set at *p* < 0.05. The comparison between the endometriosis and control groups constituted the primary analysis. The ART subanalysis, restricted to patients with endometriosis, evaluated whether ART exposure was associated with incremental risk beyond endometriosis alone. Results are presented for the composite PAS outcome, individual PAS subtypes, and maternal morbidity endpoints in Table 3 and Table 4.

For privacy protection, TriNetX suppresses exact event counts when cell sizes are fewer than 10, limiting interpretation of some rare outcomes in the ART subanalysis. To perform the analysis, TriNetX rounds up to the nearest 10, which may explain the identical confidence intervals seen in these conditions.

### 2.5. Institutional Review Board Approval and Data Compliance

Since the TriNetX database includes only deidentified patient records according to the standard in Section §164.514(a) of the HIPAA Privacy Rule, this study was exempt from full institutional review board approval. Additionally, TriNetX, LLC, complies with HIPAA, ISO 27001:2013 [14], and other relevant privacy regulations to protect health care data.

## 3. Results

### 3.1. Primary Analysis

#### 3.1.1. Incidence of PAS

**Figure 2:**
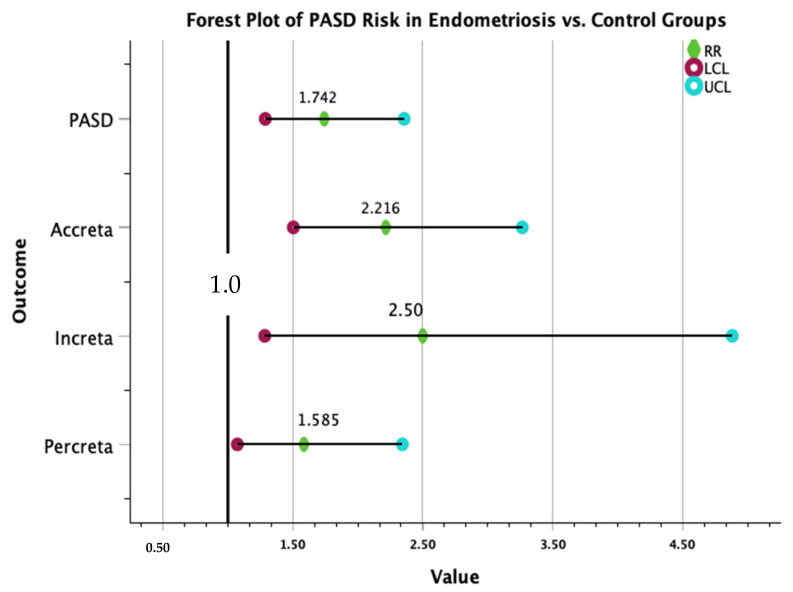
Forest plot of PAS risk in endometriosis vs. control groups.

Patients with endometriosis showed a greater overall incidence of PAS compared to matched controls (0.48% vs. 0.28%; RR = 1.74; 95% CI, 1.29–2.36; *p* = 0.0003). All PAS subtypes were also elevated: placenta accreta (0.34% vs. 0.15%; RR = 2.22), increta (0.13% vs. 0.05%; RR = 2.50), and percreta (0.27% vs. 0.17%; RR = 1.59). Complete results are available in Table 3 and Figure 2.

#### 3.1.2. Peripartum Maternal Morbidity (Endometriosis vs. Control)

In the matched cohorts, endometriosis was associated with higher rates of several maternal complications at delivery. Significant increases were observed for peripartum hysterectomy (0.95% vs. 0.55%; RR 1.72), transfusion (1.61% vs. 1.28%; RR 1.26), postpartum hemorrhage (5.79% vs. 4.29%), readmission (4.83% vs. 3.96%), wound dehiscence (0.25% vs. 0.15%; RR 1.61), critical care services (0.44% vs. 0.31%; RR 1.43), and sepsis (0.37% vs. 0.24%; RR 1.56). Differences were not significant for disseminated intravascular coagulation (DIC; 0.06% vs. 0.06%; RR 1.00), hemorrhagic shock (0.06% vs. 0.06%; RR 1.00), or uterine artery embolization (0.09% vs. 0.06%; RR 1.70). Results are displayed in Table 4.

### 3.2. Sub-Analysis

#### 3.2.1. Incidence of PAS

**Figure 3:**
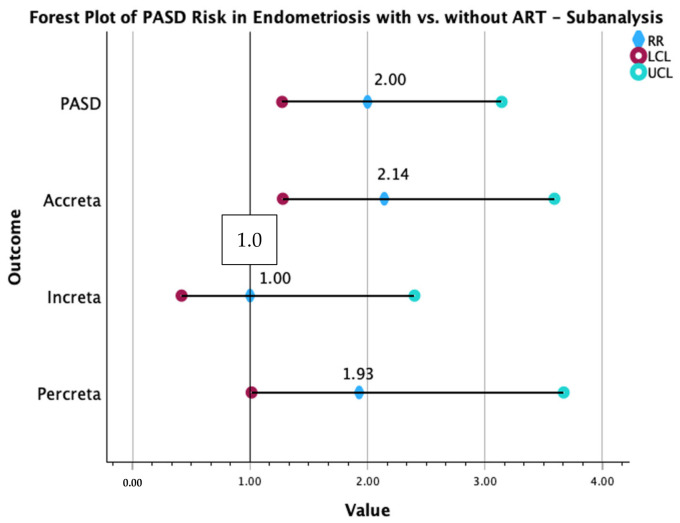
Forest plot of PAS risk in endometriosis with vs. without ART.

Among pregnancies in individuals with endometriosis, use of assisted reproductive technology (ART) did significantly increase PAS risk compared to pregnancy without a history of ART (1.34% vs. 0.67%; RR = 2.00; *p* = 0.0021). Differences for accreta (RR = 2.14; *p* = 0.003) and percreta (RR = 1.93; *p* = 0.0418) were also significant. However, the difference for increta (RR = 1.00; *p* = 1.00) was not significant. Results are displayed in Table 3 and Figure 3.

#### 3.2.2. Peripartum Maternal Morbidity (Endometriosis with ART vs. Without ART)

Among patients with endometriosis, conception via ART was linked to higher risks of peripartum hysterectomy (1.57% vs. 0.96%; RR 1.63), transfusion (3.90% vs. 1.86%; RR 2.10), postpartum hemorrhage (9.29% vs. 5.60%; RR 1.66), and readmission (5.47% vs. 4.46%; RR 1.23) compared with conception without ART. No significant differences were observed for wound dehiscence (0.27% vs. 0.27%; RR ≈ 1.00), critical care services (0.45% vs. 0.43%; RR 1.06), DIC (0.27% vs. 0.27%; RR 1.00), hemorrhagic shock (0.27% vs. 0.27%; RR 1.00), or uterine artery embolization (0.27% vs. 0.27%; RR 1.00). Sepsis events were too rare to estimate an effect (0.00% vs. 0%). Complete results appear in Table 4.

## 4. Discussion

This multi-institutional, propensity-matched cohort shows that a history of endometriosis is associated with a significantly increased risk of placenta accreta spectrum disorders (PAS), including accreta, increta, and percreta, as well as higher rates of peripartum hysterectomy, transfusion, postpartum hemorrhage, intensive care use, readmission, and sepsis. Furthermore, this study found that ART usage among patients with endometriosis has an additive effect on the risks of PAS disorders as well as peripartum complications. These results persisted after matching for available covariates that increase the risk of PAS disorders, supporting an association that measured confounders cannot entirely explain. This study’s findings expand on the existing literature which has shown that endometriosis is linked to abnormal placentation and hemorrhagic complications associated with PAS [4,9].

Several biological interconnected pathways plausibly link endometriosis to abnormal placental adherence. Modern models emphasize that the mechanism behind PAS disorders results from impaired decidualization and mechanical failure at the endometrial-myometrial interface, especially around a scar [1,2]. It is hypothesized that endometriosis can alter tissue integrity at possible implantation locations due to its characteristics of chronic inflammation, fibrosis, altered angiogenesis, and dysregulated receptivity [8]. In this study, patients with endometriosis, independent of ART, and controlled for other PAS risk factors, were associated with significantly higher risk rates overall spectrum (RR 1.74, *p* < 0.0003), and across all types, including accreta (RR = 2.22, *p* < 0.0001), increta (RR = 2.50, *p* < 0.0055), and percreta (RR = 1.56, *p* < 0.0196), when compared to the control cohort. The progression of PAS in endometriosis found in this study may be attributed to the aberrant inflammation secondary to endometriosis, but the mechanism remains unclear and requires further investigation.

This study also finds that ART is associated with increased risk of PAS disorders in patients with endometriosis. It was shown that conception through ART was linked to an increased risk, including the composite PAS endpoint (RR = 2.00, *p* = 0.0021) and specific subtypes (Accreta RR = 2.14, *p* = 0.003; Percreta RR = 1.93, *p* = 0.0418), relative to endometriosis without ART. These findings are consistent with reports that have identified in vitro fertilization (IVF) as associated with increased risk for PAS disorders beyond traditional contributors [12,15]. Additionally, Carusi et al.’s findings show that, even with adjustment for patient factors, ART-specific factors, such as frozen embryo transfer (FET), particularly with very thin endometrial measurements, and use of non-controlled ovarian hyperstimulation cycles, increase the risk for PAS disorders [12]. However, Salmanian et al. highlight in their study that while IVF is independently linked to PAS disorders, its relative clinical significance is minor compared to the presence of placenta previa and a history of cesarean delivery [15]. The proposed mechanisms of ART influencing PAS disorder development include altered implantation dynamics during embryo transfer, protocols for endometrial preparation, and placentation influenced by previous uterine instrumentation or subtle uterine defects [13].

Beyond PAS disorders, the endometriosis cohort experienced higher risk rates for major peripartum morbidity. These findings are in line with prior population-based and meta-analytic work that highlights the association between endometriosis and hemorrhagic complications and severe maternal morbidity [8,16]. These observed outcomes could be due to the chronic inflammatory fibrosis in endometriosis, which often involves dense adhesions that distort pelvic anatomy [17]. Moreover, studies on conception modes indicate that women with endometriosis, especially those conceiving via ART, face increased rates of hemorrhage-related complications and interventions, concurring with the observation of increased transfusion and readmission in the endometriosis + ART subgroup [11,12,13].

The higher operative complications observed in endometriosis further justify the consequent higher incidence of critical care use and sepsis rate. Severe hemorrhage and complex surgeries raise the risk of massive transfusions, invasive lines, and extended operative times, all of which are associated with postoperative infection, organ dysfunction, and ICU admission [18,19,20]. Collectively, these data endorse proactive hemorrhage preparedness, early involvement of anesthesiology and critical care teams, and the implementation of standardized postoperative surveillance protocols in pregnancies complicated by endometriosis, particularly when ART, placenta previa, or previous uterine surgery are present [2,17].

Clinically, this data underpins multiple actionable strategies. These strategies may include incorporating endometriosis and adenomyosis, when present, into early risk stratification alongside known PAS disorder predictors such as placenta previa, prior cesareans or uterine surgery, and advanced maternal age. Additionally, enhanced imaging is recommended for individuals at increased risk, including a targeted second-trimester ultrasound focusing on placental location, lacunae, myometrial thickness, and the uterovesical interface; and selective MRI when sonographic features raise suspicion or if the placenta overlies a scar [17]. When risk factors such as endometriosis, ART, previa, or uterine surgery accumulate, it is recommended to plan delivery at Level III/IV centers equipped with multidisciplinary PAS teams, interventional radiology, and massive transfusion protocols [17]. Finally, patients with endometriosis contemplating assisted reproductive technology (ART) should receive counseling that addresses PAS disorders and hemorrhage risks, and should be advised on modifiable factors such as single-embryo transfer when appropriate and careful monitoring of the implantation site [12,15].

## 5. Limitations

This study is not without limitations. Variables such as parity, prior cesarean delivery history, mode of delivery, gestational age at delivery, adenomyosis, endometriosis severity/stage, and treatment history were not consistently available within the TriNetX platform and therefore could not be incorporated into the propensity score model, leaving the possibility of residual confounding. Additionally, exposure and outcome ascertainment relied on ICD-10 and CPT coding within the electronic health record. Endometriosis is known to be underdiagnosed and undercoded, raising the possibility that some patients classified within the control cohort had undiagnosed endometriosis; such nondifferential misclassification would be expected to bias effect estimates toward the null and potentially underestimate the true association. Likewise, PAS outcomes were identified through administrative coding rather than direct pathological confirmation, which may introduce outcome misclassification. Furthermore, ICD-10 code N80.x does not distinguish endometriosis stage, severity, or anatomical location, preventing assessment of whether specific disease phenotypes confer differential PAS risk. Similarly, although ART exposure was identified, detailed treatment characteristics, including IVF versus IUI and fresh versus frozen embryo transfer cycles, were not consistently available and could not be evaluated. Surveillance bias may also have been present, as patients with endometriosis and those undergoing ART are often monitored more closely during pregnancy, potentially increasing the likelihood of PAS detection. Outcomes were limited to the 7-day perinatal period captured within the study design, which may not fully capture later postpartum complications. Finally, although post-matching standardized mean differences for measured covariates were all <0.01, suggesting excellent balance, this likely reflects the exceptionally large pool of eligible controls available for matching rather than overmatching, as the propensity model included baseline demographic and clinical confounders rather than intermediary variables on the causal pathway.

## 6. Conclusions

In this study, a history of endometriosis was associated after propensity score matching for available covariates with a higher risk of placenta accreta spectrum disorders, including accreta, increta, and percreta, as well as increased peripartum morbidity. Among patients with endometriosis, conception through assisted reproductive technology conferred additional risk for several outcomes, suggesting potential synergy between an altered uterine environment and ART-related implantation processes. These results support including endometriosis (and ART exposure) in early risk assessment, improving prenatal imaging and delivery planning at specialized centers, and standardizing hemorrhage preparedness for this vulnerable population. Recognizing endometriosis is associated with increased risk allows for more personalized care plans, potentially improving both short and long-term outcomes for these patients.

## Figures and Tables

**Table 1:** Demographic characteristics of pre-matched and post-matched cohort patients with and without a history of endometriosis.

Demographics Characteristics								
Variable	Endometriosis (Pre)	Control (Pre)	*p* (Pre)	Std Diff (Pre)	Endometriosis (Post)	Control (Post)	*p* (Post)	Std Diff (Post)
Age at Index (mean ± SD)	30.7 ± 6.3	28.5 ± 6.6	<0.0001	0.3347	30.6 ± 6.3	30.7 ± 6.5	0.4226	0.0084
Female (%)	97.70%	98.30%	<0.0001	0.0414	97.80%	97.60%	0.6282	0.0051
Unknown Gender (%)	2.30%	1.70%	0.0001	0.0414	2.30%	2.30%	0.6282	0.0051
Hispanic or Latino (%)	11.96%	21.07%	<0.0001	0.2473	11.97%	11.75%	0.5184	0.0067
Not Hispanic or Latino (%)	71.89%	54.18%	<0.0001	0.3728	71.86%	72.24%	0.4288	0.0083
White (%)	63.96%	54.42%	<0.0001	0.1951	63.88%	64.19%	0.6476	0.0048
Black or African American (%)	11.83%	15.87%	<0.0001	0.1171	11.87%	11.87%	0.7583	0.0032
Asian (%)	8.63%	6.35%	<0.0001	0.0868	8.65%	8.66%	0.9112	0.0012
Other Race (%)	3.80%	6.51%	<0.0001	0.1226	3.81%	3.89%	0.5272	0.0066
American Indian/Alaska Native (%)	0.35%	0.50%	0.0039	0.0232	0.35%	0.31%	0.5239	0.0067

**Table 2:** Comorbidities of pre-matched and post-matched cohort patients with and without a history of endometriosis.

Comorbidities								
Variable	Endometriosis (Pre)	Control (Pre)	*p* (Pre)	Std Diff (Pre)	Endometriosis (Post)	Control (Post)	*p* (Post)	Std Diff (Post)
Nicotine Dependence (%)	7.24%	3.52%	<0.0001	0.1654	7.24%	7.34%	0.7028	0.0081
Placenta Previa (%)	5.45%	3.40%	<0.0001	0.1189	5.44%	5.42%	0.417	0.0085
Hypertensive Disorders (%)	5.33%	3.10%	<0.0001	0.1399	5.26%	5.32%	0.7776	0.009
Leiomyoma (%)	5.25%	1.54%	<0.0001	0.206	5.21%	5.17%	0.9251	0.0092
Diabetes Mellitus (%)	2.50%	1.72%	<0.0001	0.0546	2.48%	2.44%	0.7622	0.0032
Procedures								
Dilation and curettage	2.32%	0.10%	<0.0001	0.2044	2.29%	2.23%	0.4964	0.0064
Hysteroscopy	0.19%	0.02%	<0.0001	0.0534	0.19%	0.17%	0.9134	0.0011
Body Mass Index (mean ± SD)								
BMI 20–25 kg/m (%)	26.96%	17.63%	<0.0001	0.2693	30.59%	28.94%	0.9449	0.0049
BMI 25–30 kg/m (%)	37.57%	25.26%	<0.0001	0.2676	34.83%	34.57%	0.9227	0.0013
BMI 30–35 kg/m (%)	26.46%	19.00%	<0.0001	0.1787	26.44%	26.33%	0.9245	0.0005
BMI 35–40 kg/m (%)	13.96%	9.96%	<0.0001	0.1188	13.99%	13.95%	0.9437	0.0003
BMI > 40 kg/m (%)	6.28%	6.28%	0.0663	0.0663	6.28%	6.26%	0.9539	0.0005

**Table 3:** PAS disorders composite and by subtype (accreta, increta, percreta) in 1:1 propensity-matched patients with endometriosis vs. control and endometriosis and ART vs. endometriosis without ART use.

Endometriosis vs. Control					
Condition	% of Outcomes Endo	% of Outcomes Control	Risk Ratio RR	95% Confidence Interval (CI)	*p*-Value
Bolded = significant					
**PAS Disorders**	**0.48%**	**0.28%**	**1.74**	**(1.288, 2.357)**	**0.0003**
**Placenta Accreta**	**0.34%**	**0.15%**	**2.22**	**(1.504, 3.266)**	**<0.0001**
**Increta**	**0.13%**	**0.05%**	**2.50**	**(1.28, 4.882)**	**0.0055**
**Percreta**	**0.27%**	**0.17%**	**1.59**	**(1.073, 2.343)**	**0.0196**
Sub-analysis: Endometriosis and ART vs. Endometriosis without ART					
Condition	% (n) of outcomes Endo + ART	% (n) of outcomes Endo − ART	Risk Ratio RR	95% confidence Interval (CI)	*p*-value
Bolded = significant					
**PAS Disorders**	**1.34%**	**0.67%**	**2.00**	**(1.273, 3.142)**	**0.0021**
**Placenta Accreta**	**1.08%**	**0.50%**	**2.14**	**(1.279, 3.591)**	**0.003**
Increta *	<10	<10	1.00	(0.417, 2.402)	1
**Percreta**	**0.65%**	**0.34%**	**1.93**	**(1.013, 3.672)**	**0.0418**

PAS = Placenta Accreta Spectrum Disorder. * = The patient count is too small.

**Table 4:** Complications in 1:1 propensity-matched patients with endometriosis vs. control and endometriosis and ART vs. endometriosis without ART use.

Endometriosis vs. Control					
Condition	% of Outcomes Endo	% of Outcomes Control	Risk Ratio RR	95% Confidence Interval (CI)	*p*-Value
Bolded = significant					
**Peripartum Hysterectomy**	**0.95%**	**0.55%**	**1.72**	**(1.381, 2.139)**	**<0.0001**
**Transfusion**	**1.61%**	**1.28%**	**1.26**	**(1.083, 1.469)**	**0.0028**
**PPH**	**5.79%**	**4.29%**	**1.35**	**(1.244, 1.462)**	**<0.0001**
**Readmission**	**4.83%**	**3.96%**	**1.22**	**(1.106, 1.34)**	**<0.0001**
**Wound Dehiscence**	**0.25%**	**0.15%**	**1.61**	**(1.003, 2.575)**	**0.0464**
**Critical Care Services**	**0.44%**	**0.31%**	**1.43**	**(1.016, 2.009)**	**0.0392**
DIC *	<10	<10	1.00	(0.416, 2.402)	1
Hemorrhagic Shock *	<10	<10	1.00	(0.416, 2.402)	1
Uterine Artery Embolization	0.09%	0.06%	1.70	(0.779, 3.712)	0.1778
**Sepsis**	**0.37%**	**0.24%**	**1.56**	**(1.115, 2.192)**	**0.0089**
Sub-analysis: Endometriosis and ART vs. Endometriosis without ART					
Condition	% of outcomes Endo + ART	% of outcomes Endo − ART	Risk Ratio RR	95% confidence Interval (CI)	*p*-value
Bolded = significant					
**Peripartum Hysterectomy**	**1.57%**	**0.96%**	**1.63**	**(1.082, 2.467)**	**0.0183**
**Transfusion**	**3.90%**	**1.86%**	**2.10**	**(1.586, 2.781)**	**<0.0001**
**PPH**	**9.29%**	**5.60%**	**1.66**	**(1.407, 1.956)**	**<0.0001**
**Readmission**	**5.47%**	**4.46%**	**1.23**	**(1.005, 1.496)**	**0.0438**
Wound Dehiscence *	<10	<10	1.00	(0.417, 2.402)	1
Critical Care Services	0.45%	0.43%	1.06	(0.538, 2.100)	0.8615
DIC *	<10	<10	1.00	(0.417, 2.402)	1
Hemorrhagic Shock *	<10	<10	1.00	(0.417, 2.402)	1
Uterine Artery Embolization *	<10	<10	1.00	(0.417, 2.402)	1
Sepsis	0.00%	0.00%	-	-, -	-

* = The patient count is too small.

## Data Availability

Data can be found on TriNetX Research Network.
